# Combined Effects of Soil Biotic and Abiotic Factors, Influenced by Sewage Sludge Incorporation, on the Incidence of Corn Stalk Rot

**DOI:** 10.1371/journal.pone.0155536

**Published:** 2016-05-13

**Authors:** Raquel Ghini, Nara Lúcia Perondi Fortes, Juan A Navas-Cortés, Carlos Alberto Silva, Wagner Bettiol

**Affiliations:** 1 Embrapa Environment, Jaguariúna, SP, Brazil; 2 Taubaté University, Taubaté, SP, Brazil; 3 Instituto de Agricultura Sostenible, Consejo Superior de Investigaciones Científicas, Córdoba, Spain; 4 Department of Soil Science, Federal University of Lavras, Lavras, MG, Brazil; National University of Ireland—Galway, IRELAND

## Abstract

The objectives of this study were to evaluate the combined effects of soil biotic and abiotic factors on the incidence of Fusarium corn stalk rot, during four annual incorporations of two types of sewage sludge into soil in a 5-years field assay under tropical conditions and to predict the effects of these variables on the disease. For each type of sewage sludge, the following treatments were included: control with mineral fertilization recommended for corn; control without fertilization; sewage sludge based on the nitrogen concentration that provided the same amount of nitrogen as in the mineral fertilizer treatment; and sewage sludge that provided two, four and eight times the nitrogen concentration recommended for corn. Increasing dosages of both types of sewage sludge incorporated into soil resulted in increased corn stalk rot incidence, being negatively correlated with corn yield. A global analysis highlighted the effect of the year of the experiment, followed by the sewage sludge dosages. The type of sewage sludge did not affect the disease incidence. A multiple logistic model using a stepwise procedure was fitted based on the selection of a model that included the three explanatory parameters for disease incidence: electrical conductivity, magnesium and *Fusarium* population. In the selected model, the probability of higher disease incidence increased with an increase of these three explanatory parameters. When the explanatory parameters were compared, electrical conductivity presented a dominant effect and was the main variable to predict the probability distribution curves of Fusarium corn stalk rot, after sewage sludge application into the soil.

## Introduction

Organic amendments have been used as a strategy for the management of soil borne pathogens based on their capacity to induce suppressiveness [1, 2]. Suppressive soils are defined as those in which disease development is suppressed by biotic and/or abiotic factors even though a virulent pathogen and susceptible host are present [3]. However, amendment application can also increase disease incidence, inducing the soil to become conducive to disease development. Bonanomi et al. [4] found that organic matter amendments were suppressive in 45% of the studied cases, no significant changes occurred in 35%, and disease incidence increased in 20% of the cases.

Sewage sludge is a widely available organic matter amendment that is rich in macro- and micronutrients. The agricultural and forestry disposal of sewage sludge can modify soil biotic and abiotic characteristics, for example, the dynamics of microbial activity, organic matter decomposition, nutrient cycling and pest and disease severity [5–8]. However, studies on sewage sludge are mainly conducted to assess the nutritional effects and issues related to heavy metal accumulation in soils. The lack of knowledge on the possible effects of sewage sludge on plant pathogens is one of the problems related to its use in agriculture. Soil incorporation of sewage sludge reduced the incidence or severity of Sclerotinia lettuce drop caused by *Sclerotinia minor* [9]; rot and bean seedling damping-off caused by *Sclerotium rolfsii* [5, 7]; root rot on sugarcane roots caused by *Pythium arrhenomanes* [10]; root rot on peppers caused by *Phytophthora capsici* [11]; citrus damping-off caused by *Phytophthora nicotianae* [12] and damping-off of peas and cotton caused by *Rhizoctonia solani* and *Pythium ultimum* [13]. An induced suppressiveness was also observed for Fusarium wilt on cucumber, caused by *Fusarium oxysporum* f. sp. *cucumerinum* [14]; on chrysanthemum, caused by *Fusarium oxysporum* f. sp. *chrysanthemi* [15] and on tomato, caused by *Fusarium oxysporum* f. sp. *lycopersici* [16]. However, Chellemi et al. [17] and Ghini et al. [7] observed no effect of sewage sludge on soil suppressiveness to bacterial wilt and *Fusarium oxysporum* f. sp. *lycopersici* on tomato plants, respectively. There are reports of increased diseases because of sewage sludge incorporation, for example, Gibberella rot on corn [18], and root rot of beans, peas and cotton caused by *Pythium ultimum* and *Thielaviopsis basicola* [19]. The organic amendments incorporated to the soil can be suppressive or conducive to plant pathogens by affecting soil biological, chemical and physical characteristics. The mechanisms are directly due to the presence of toxic compounds (volatile fatty acid, nitrous acid, and ammonia), and indirectly by the stimulation of soil microorganisms [10, 20–23]. The factors that determine the effects of soil incorporation of sewage sludge on soil suppressiveness have to be identified to allow the safe use of this waste in agriculture.

In general, these studies mentioned above were conducted under temperate climate conditions and performed over short-term periods. However, an interdisciplinary and long-term field study was conducted under tropical conditions to evaluate the environmental impacts of two types and five dosages of sewage sludge [24]. The soil chemical, physical and biological properties, and corn growth, pests, diseases and yield were evaluated. During the second and third year of this research, Bettiol [25] observed that the incidence of corn stalk rot caused by *Fusarium* spp. was positively correlated with the dosage of sewage sludge incorporated into the soil.

Corn stalk rot is caused by *Fusarium* spp., mainly *F*. *verticillioides*, *F*. *proliferatum*, *F*. *subglutinans*, *F*. *graminearum* and *F*. *temperatum* [26–28], which affect roots, the plant base, internodes, kernels and ears. The disease causes reduced plant growth and rotten stalks and leaf sheaths. In mature plants, it causes a whitish-pink to salmon discoloration of the internal stalk pith tissues. The pathogen survives in crop residues on the soil and can be transmitted by seeds [29, 30]. High soil moisture and temperatures of approximately 28–30°C are favorable conditions for the disease [28].

The objectives of this study were to evaluate the combined effects of soil biotic (fungal, bacterial and *Fusarium* spp. populations) and abiotic [pH, organic matter (OM), P, K, Ca, Mg, H+Al, sum of bases (SB), cation exchange capacity (CEC), base saturation (V), electrical conductivity (EC), N-NH_4_^+^ and N-NO_3_^-^] factors on the incidence of corn stalk rot during four annual incorporations of sewage sludge into soil in a 5-years field assay and to predict the effects of these variables on corn stalk rot.

## Materials and Methods

### Field experiment

A 5-years field experiment was carried out at Embrapa Environment, located in Jaguariúna, São Paulo State, Brazil (latitude 22°41’S, longitude 47°W, altitude of 570 m a.s.l.). The climate is humid subtropical (Cfa according to the Köppen classification) with hot rainy summers (mean air temperature = 22.6°C, mean precipitation = 663 mm) and cold dry winters (mean air temperature = 16.9°C, mean precipitation = 98 mm) [31]. The soil at the experimental area was a dark red distroferric latosol (clayey texture) which physical and chemical characteristics in the 0–20 cm layer, before the onset of the study follows: pH in water = 5.8; OM = 25.5 g/kg; P = 3.5 mg/cm^3^; K = 1.51, Ca = 27.5, Mg = 8.5, Al = 1, H = 35, and CEC = 73.5 mmol_c_/dm^3^; SB = 50.8%, and clay = 450 g/kg [24].

Sewage sludges were obtained from wastewater treatment plants located in Barueri, São Paulo State, which treats domestic and industrial sewage, and in Franca, São Paulo State, which treats only domestic sewage. Both plants use the activated sludge process. On average, the characteristics of Barueri sewage sludge were C = 340.1, N = 48.6, P = 1.3, Ca = 30.1, Mg = 3.7, S = 13.5 g/kg; Cu = 921.5, Zn = 3037.2, Fe = 39165.8, Mn = 341.6, B = 21 mg/kg; pH = 7.1; humidity = 75.2% and volatile solids = 55.5%; and Franca sewage sludge were C = 381.8, N = 59.9, P = 1.1, Ca = 19.1, Mg = 2.9, S = 12.9 g/kg; Cu = 244.6, Zn = 1136.3, Fe = 38892.8, Mn = 400.3, B = 18.3 mg/kg; pH = 7.2; humidity = 80.2% and volatile solids = 64.7%. Bettiol and Ghini [24] described the characteristics of these two types of sewage sludge and the amounts of sludge and fertilizers applied annually in each treatment.

For each sewage sludge, Franca (F) and Barueri (B), the following treatments were used: control with mineral fertilization (NPK) recommended for corn (N = 90 kg/ha, P_2_O_5_ = 90 kg/ha and K_2_O = 70 kg/ha); control without fertilization (0N); applied dosage of sewage sludge based on the N concentration that provides the same amount of N as the mineral fertilizer treatment (1N); and two (2N), four (4N) and eight (8N) times the recommended N dosage for the corn crop. The calculation of the sewage sludge rates was performed as a function of the N available for plants considering the N mineralization rate was 30%. Barueri sludge amounts applied to obtain 1N treatment was 8095, 3995, 5315, 5295 and 3200 kg/ha dry matter, and Franca sludge 3014, 3504, 3766, 4432 and 4300 kg/ha dry matter, for the first, second, third, fourth and fifth years of the sewage sludge application, respectively. Supplementary K was applied in the sewage sludge treatments when necessary [24]. The sewage sludge was incorporated annually over five consecutive years. It was toss distributed over the total area of the plots and incorporated to a depth of 20 cm using a rotary harrow, 3 or 4 days before sowing.

After the sewage sludge application, the corn variety CATI AL30 (minicrop) was cultivated in the first year; hybrid AG1043 in the second year; and in the third, fourth and fifth years hybrid Savanna 133S was sown (6–7 seeds per meter within a row). In the third cultivation, the pH of each plot was corrected to pH 5.7, with incorporation of dolomitic lime, one month before the sludge application, based on a soil-neutralizing curve.

The agricultural practices adopted were those traditionally used locally for the crop without irrigation. The stubble was removed from the plots before sludge application.

The experiment was set up as a repeated measures design with year of application as between subject factor, sewage sludge and dose of application as within-subject factors and three replications as subject factor. Each plot measured 10 x 20 m, with 12 rows per plot. Hedgerows separated plots with at least 5 m on each side. In this study, assessments were performed in the second, third, fourth and fifth years of the sewage sludge application and are referred as years 1, 2, 3 and 4, respectively.

### Soil chemical analyses

Soil samples (0 to 20 cm) were collected 90 days after the sewage sludge application. At least five composited soil samples were collected per plot for chemical and biological analyses. The soil samples were analyzed to determine pH in water, OM, P, K, Ca, Mg, H+Al, SB, CEC, V and EC [32, 33]. In years 3 and 4, N-NH_4_^+^ and N-NO_3_^-^ were also analyzed [34].

### Soil microbial populations

The cultivable microbial populations in the soil were determined using the serial-dilution method followed by plating on a selective culture medium. Thornton [35] and Nash and Snyder [36] media were used to estimate the bacterial and *Fusarium* spp. populations, respectively. In years 3 and 4, Martin media [37] was used to estimate fungal population. Aliquots (0.1 mL) of the dilutions of each soil sample were transferred to each culture medium in Petri dishes; three replicates were used. The plates were incubated at 28°C in the dark for 3 to 6 days. Assessments were performed by counting the number of colonies per Petri dish, expressed as colony-forming units/g of dry soil (CFU/g dry soil). Microscopic examination was used to identify *Fusarium* spp. based on the morphology of their spore structures.

### Disease incidence

The incidence of diseased plants was evaluated approximately 90 days after sowing. Specifically, the number of plants showing symptoms of corn stalk rot caused by *Fusarium* spp. in the central row of the plots were counted.

In years 3 and 4, in addition to the evaluation of the symptoms, pathogen isolation was performed to estimate the incidence of *Fusarium* colonization. Segments of roots and stalks (3 to 5 mm; 25 segments per plant; 6 plants per plot) were rinsed with tap water for 30 min., immersed in sodium hypochlorite 1% (v/v) for 1 min., and then rinsed in sterile distilled water. The disinfested plant segments were transferred to PDA supplemented with oxytetracycline and incubated at 25 ± 2°C under a 12-h photoperiod. The percentage of *Fusarium-*colonized segments was assessed using microscopic inspection. In the same plants, the percentage of ears with *Fusarium* rot symptoms was also evaluated. Isolations from symptomatic roots and stalks consistently yielded *Fusarium* sp. identified by morphological characteristics. A selection of eight isolates based on similarity of the isolates in colony morphology and morphometric fungal structures were identified by molecular methods. The translation elongation factor-1*α* (TEF-1 *α*) gene was amplified using primers EF1 and EF2 [38] and the fragments obtained were subsequently sequenced in both directions following procedures described in Jiménez-Fernández et al. [39]. Sequences were deposited in the NCBI Sequence Database (Accession No. KU372137 to KU372144). A BLAST search was done against sequences in the FUSARIUM-ID v.1.0 database (http://fusarium.cbio.psu.edu) [40] and the GenBank database that revealed 100% sequence identity to *F*. *verticillioides*.

### Yield

Corn yield was evaluated by harvesting the ears from the six central rows of each plot. The harvested ears were dried under ambient conditions, the grains were weighed, and yield was expressed as kg/ha.

### Data analyses

Firstly, the overall effects of experimental treatment combinations on the incidence of corn stalk disease and corn yield as well as on soil biotic (bacterial and *Fusarium* spp. populations) and abiotic [pH, OM, P, K, Ca, Mg, H+Al, SB, CEC, V and EC] parameters was explored using analysis of variance (ANOVA) to determine the extent of explained variability due to main factors in the study (i.e., sewage sludge origin and dose) and how these effects are consistent thorough the 5-years of the study using the General lineal model procedure of SAS software (version 9.3; SAS Institute, Cary, NC, USA). Later on cluster analyses were used to establish functional groups of correlated experimental treatments. For that, the agglomerative clustering based on the Spearman correlation matrix was performed using the Ward clustering method [41]. A heat map was developed to visualize the values of the different treatments and parameters used in the analysis. All cluster analysis calculations were performed using the R software, version 3.0.2 (R Foundation for Statistical Computing, http://www.R-project.org/) with the *cluster* [42], *gplots* [43] and *vegan* [44] packages.

The relationship between the classes of corn stalk disease severity and the stress-related parameters was estimated using logistic regression analysis. Logistic models are direct probability models that are stated in terms of the probability of the occurrence of an event (i.e., disease incidence class) under a given set of conditions (i.e., biotic or abiotic parameters) [45]. In this study, a multinomial logistic regression model was fitted to each biotic and abiotic parameter as explanatory variables, and disease incidence class was used as the response variable, using the lowest disease incidence class as the reference category [46]. Three disease incidence homogenous groups with low, moderate and high disease incidence were determined using the FASTCLUS procedure of SAS using the nearest centroid sorting algorithm in which observations were classified according to their disease incidence values. To assess the statistical significance of each explanatory variable, each model was compared with the null model using a likelihood ratio test. The proportion of the variance explained by each model was evaluated using the maximum rescaled *R*^2^ coefficient of determination and the classification accuracy. This was performed using the LOGISTIC procedure of SAS software. To assess the combined effects of all biotic and abiotic factors, a multiple logistic regression model was fitted using the stepwise procedure that allowed selecting only the biotic or abiotic factors with a significant contribution to the model.

## Results

**Fig 1:**
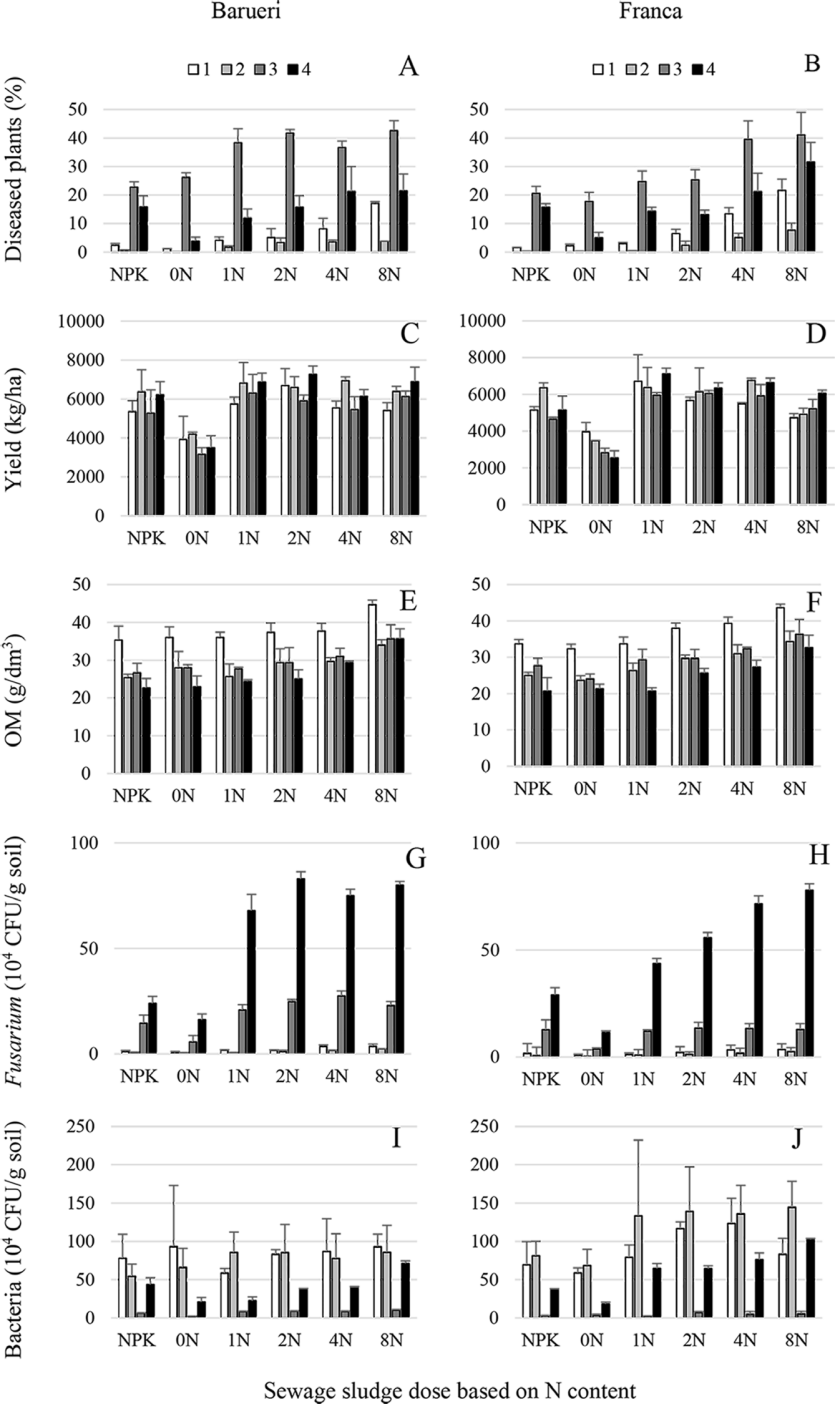
Incidence of Fusarium corn stalk rot, yield, organic matter content, *Fusarium* and bacterial populations in soil amended with two types of sewage sludge. (A, B) Corn stalk rot incidence, (C, D) corn yield, (E, F) organic matter content (OM), (G, H) *Fusarium* and (I, J) bacterial populations in soil treated with sewage sludge from Franca (A, C, E, G, I) and Barueri (B, D, F, H, J) at different dosages [0N, 1N, 2N, 4N and 8N, based on the N concentration that provided the same amount of N as the mineral fertilizer (NPK) recommended for corn] for four years (1, 2, 3 and 4). Error bars represent the SD of three replicates.

**Fig 2:**
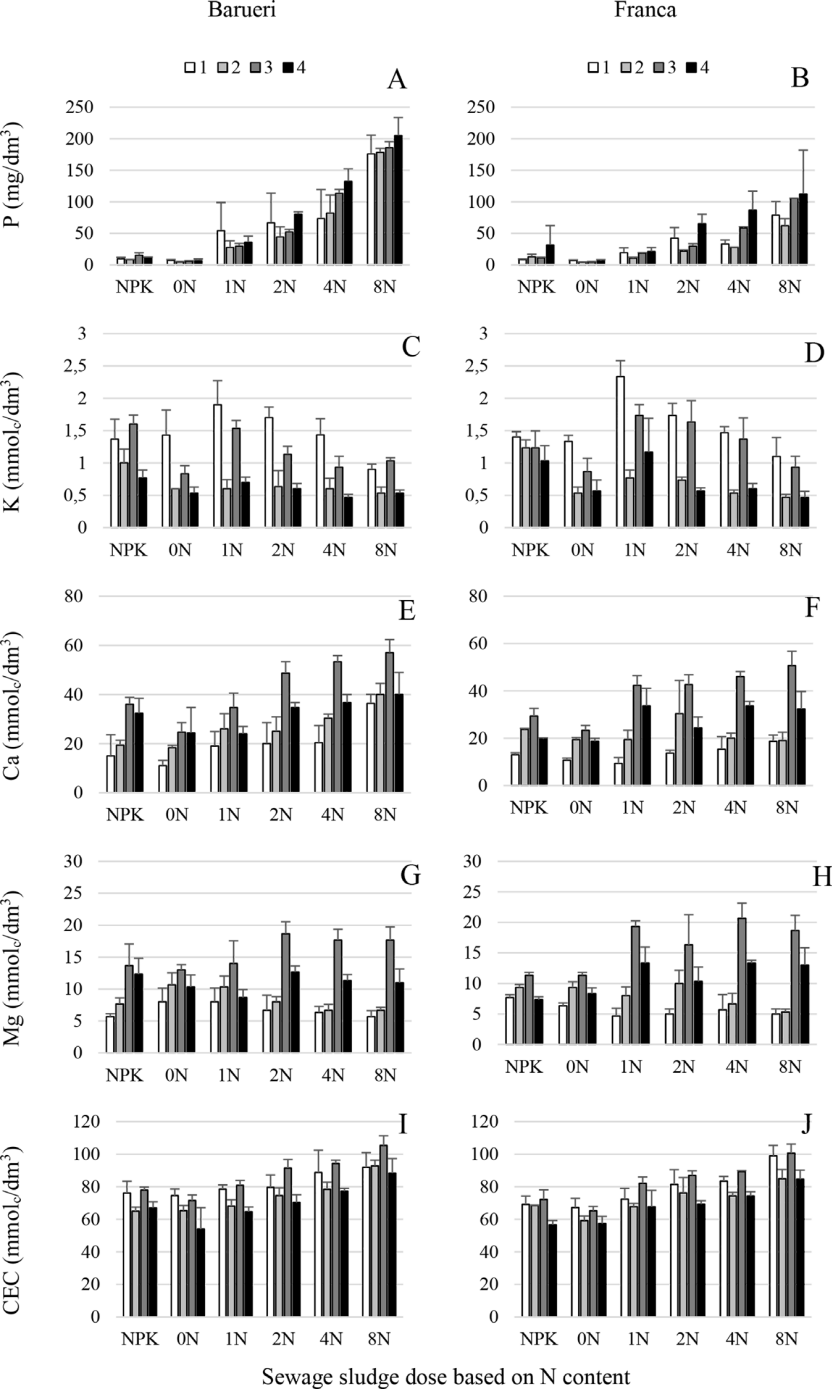
Chemical attributes (P, K, Ca, Mg and CEC) of soil amended with two types of sewage sludge. (A, B) Phosphorus, (C, D) potassium, (E, F) calcium, (G, H) magnesium contents and (I, J) cation exchange capacity (CEC) of soil treated with sewage sludge from Franca (A, C, E, G, I) and Barueri (B, D, F, H, J) at different dosages [0N, 1N, 2N, 4N and 8N, based on the N concentration that provided the same amount of N as the mineral fertilizer (NPK) recommended for corn] for four years (1, 2, 3 and 4). Error bars represent the SD of three replicates.

**Fig 3:**
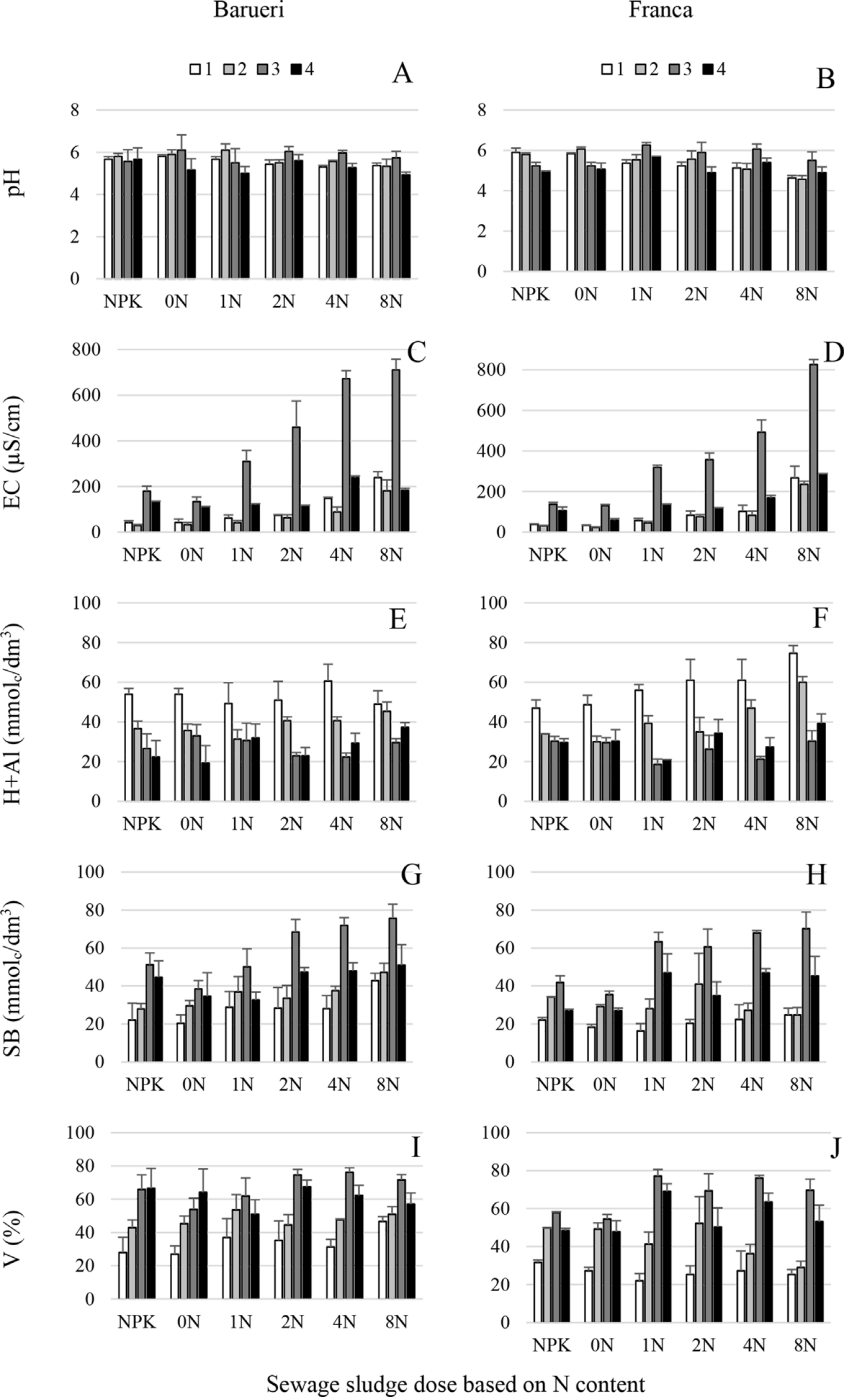
Chemical attributes (pH, EC, H+Al, SB, V) of soil amended with two types of sewage sludge. (A, B) pH, (C, D) electrical conductivity (EC), (E, F) hydrogen and aluminum content (H+Al), (G, H) sum of bases (SB) and (I, J) base saturation (V) of soil treated with sewage sludge from Franca (A, C, E, G, I) and Barueri (B, D, F, H, J) at different dosages [0N, 1N, 2N, 4N and 8N, based on the N concentration that provided the same amount of N as the mineral fertilizer (NPK) recommended for corn] for four years (1, 2, 3 and 4). Error bars represent the SD of three replicates.

Overall, biotic parameters were significantly influenced (*P*<0.05) by year of the experiment, source and dosage of sewage sludge, and their interactions (S1 Table, Fig 1). Stalk rot incidence tended to increase over time, to reach a maximum 31.42% in the year 3, being negatively correlated with corn yield that showed maximum values of 5943.7 kg/ha in year 2 (Fig 1A–1D), as well as with *Fusarium* and bacterial populations that showed their maximum values of 118.67 x 10^4^ and 96.43 x 10^4^ CFU/g, respectively, in year 2 (Fig 1G–1J). Type of sewage sludge did not influenced significantly (*P*≥0.05) biotic parameters except for corn yield and bacterial population that showed higher values (*P*<0.05) in Franca and Barueri, respectively (S1 Table, Fig 1). Increasing the dosage of sewage sludge that were incorporated into the soil resulted in an increase in corn stalk rot, and in *Fusarium* and bacterial populations that were significantly higher (*P*<0.05) in treated plots compared to that either non treated or those with mineral fertilization (S1 Table, Fig 1). This effect was true for both sewage sludge types except for corn stalk rot incidence that showed higher values (*P*<0.05) for Barueri at the lower dosages of 1N and 2N, but significantly higher (*P*<0.05) for Franca with the highest dosage 8N (S1 Table, Fig 1). In any case, high sludge dosages did not result in increased corn yield, mainly because of increased disease incidence and nutritional imbalance resulting from cumulative applications (Figs 1–3).

Abiotic parameters showed clearly distinct patterns over time (Figs 1E, 1F, 2 and 3). A decrease during the four years of experiments was observed for OM, K and H+Al, but the opposite occurred for P. The remaining parameters reached their maximum values on year 3 to decrease to minimum values on year 1 (Ca, Mg, SB and V), year 2 (EC) or year 4 (pH and CEC) (S2 and S3 Tables, Figs 2 and 3). All abiotic parameters in the study were significantly influenced (*P*<0.05) by sewage sludge source and dosage, except for OM, EC and Mg that showed no significant differences (*P*≥0.05) due to either sewage sludge types or dosage (Mg). Moreover a significant interaction (*P*<0.05) between both experimental factors was detected for EC, H+Al, P and Ca (S2 and S3 Tables, Figs 1–3). Sewage sludge origin did not determine significant differences (*P*≥0.05) for OM, Mg and EC, but significantly higher (*P*<0.05) values for P, Ca, CEC, pH, SB, and V were reached when Barueri sewage sludge was applied compared to Franca, and the opposite occurred for K and H+Al (S2 Table, Figs 1–3). Dosage of the sewage sludge amendment also had an effect on the estimated abiotic parameters. Thus, for both types of sewage sludge, OM, CEC and SB, increased with the increase in dosage of the amendment, but K, pH and V showed a negative trend with the increase in the dosage (Figs 1–3). Due to the significant sewage sludge x dosage interaction (*P*<0.05), P, Ca, EC and H+Al showed distinct dosage patterns for each sewage sludge type (S2 and S3 Tables, Figs 1–3).

**Fig 4:**
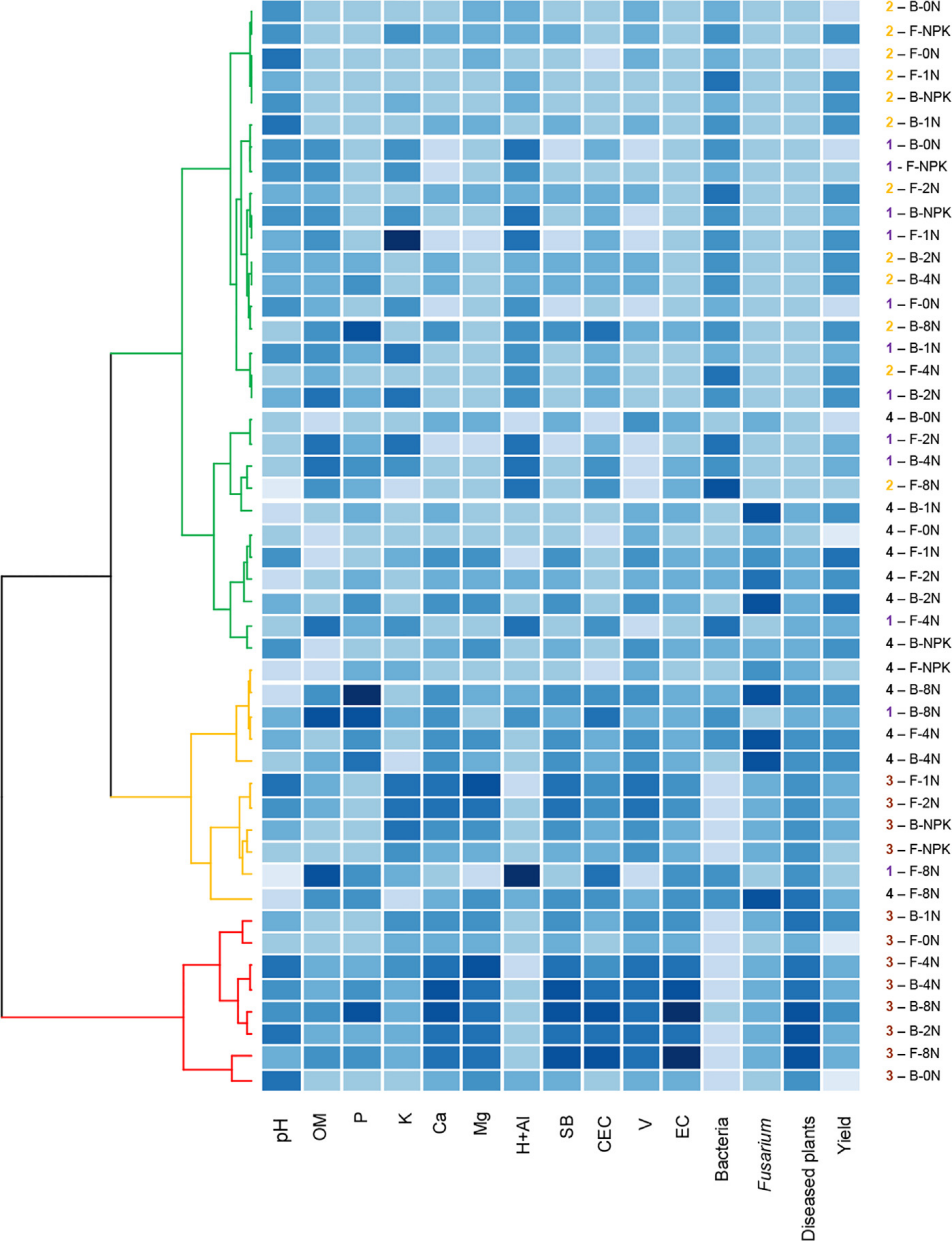
Dendrogram showing results of cluster analyses and heat map representation of disease, yield, and soil abiotic and biotic parameters amended with two types of sewage sludge. Dendrogram showing the results of the cluster analyses (red clusters represent high disease incidence, orange clusters represent intermediate incidence and green clusters represent low disease incidence) and a heat map representation of corn stalk rot incidence and yield and their relationship with soil abiotic (pH, OM, P, K, Ca, Mg, H+Al, SB, CEC, V, EC) and biotic parameters (bacterial and *Fusarium* populations) of soil treated with sewage sludge from Franca (F) and Barueri (B) at different dosages [0N, 1N, 2N, 4N and 8N, based on the N concentration that provided the same amount of N as the mineral fertilizer (NPK) recommended for corn] for years 1 (purple), 2 (orange), 3 (brown) and 4 (black). The blue intensity represents the mean value of the variable (higher values are represented by darker blue shades).

To better deciphering the complex interactions between Fusarium stalk rot and corn yield with the 13 soil parameters [11 abiotic (pH, OM, P, K, Ca, Mg, H+Al, SB, CEC, V and EC) and 2 biotic parameters (bacterial and *Fusarium* populations)], a multivariate hierarchical cluster analysis was performed and the variables were ordered by disease incidence (Fig 4). This global analysis confirmed results of ANOVA analyses highlighting a clear effect of the year of the experiment, followed by the sewage sludge treatments, i.e., sludge dosages (Fig 4). Overall, high values of disease incidence were related to EC, V, CEC, SB, Mg, Ca and the *Fusarium* population. Low disease incidence was related to high values of the bacterial population and H+Al (Fig 4). Separate analyses of the results for each year of the experiment indicated that sludge dosages of 8N and 4N for both types of sludge resulted in a higher incidence of diseased plants (S1 Fig).

In years 3 and 4, in addition to the increased disease incidence, higher sludge dosages resulted in a higher frequency of pathogen isolation from rotten stalks and symptomatic ears. The fungal population and the N-NH_4_^+^ and N-NO_3_^-^ contents were also related to high sewage sludge dosages and disease incidence. The cumulative effect of the sewage sludge applications was particularly evident in years 3 and 4 when almost all analyzed variables had the lowest values in response to the 0N dosage of either the Barueri or the Franca sludge (S1 Fig).

Three distinct groups of disease incidence were observed, ranging from low disease incidence (0 to 9%, class 1) to moderate (10 to 27%, class 2) and high incidence (28 to 50%, class 3). A direct relationship was observed between the disease incidence group and the soil parameters P, Ca, Mg, SB, CEC, V, EC, N-NH_4_^+^, N-NO_3_^-^, as well as with Fusarium stalk rot and symptomatic ears (S2 Fig).

**Fig 5:**
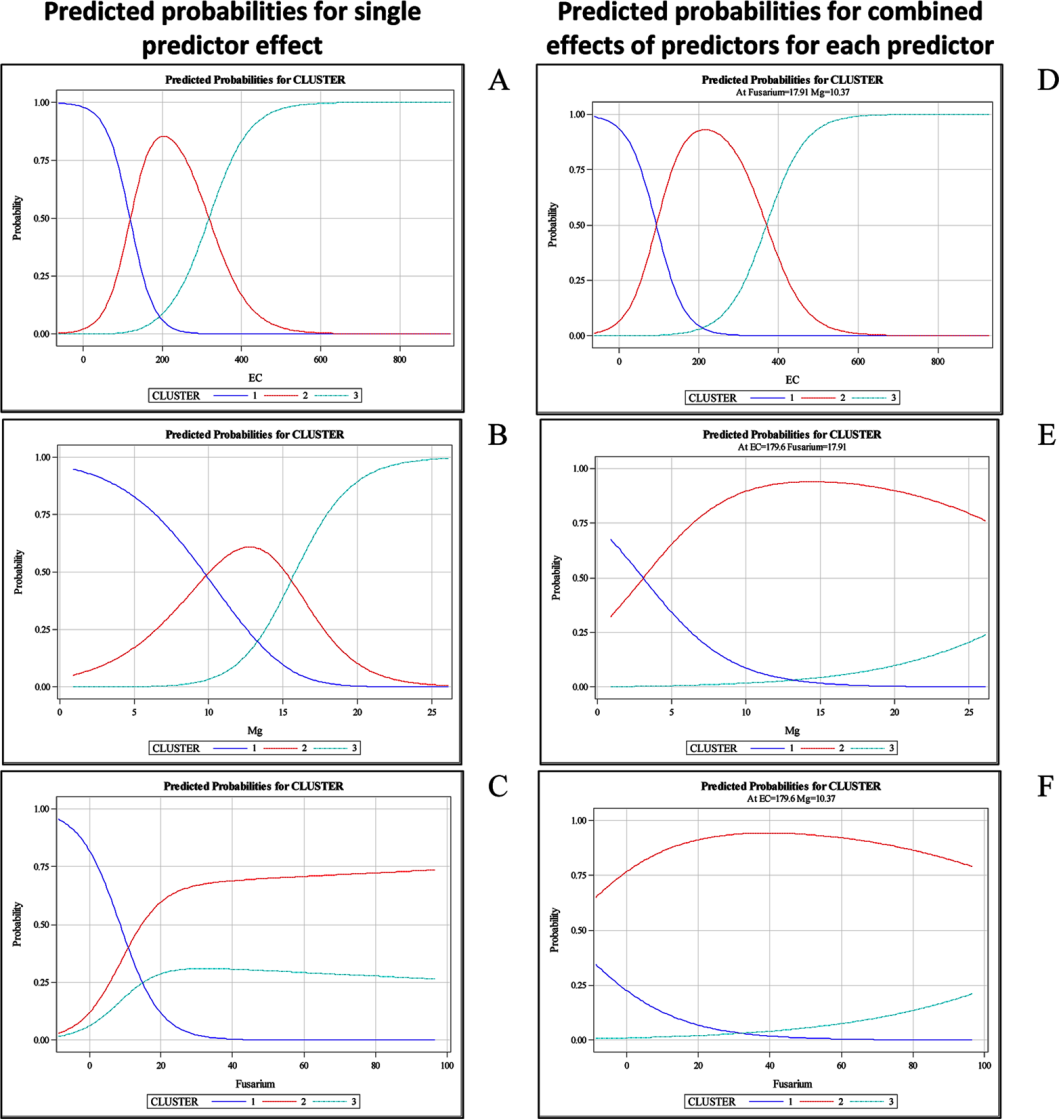
Predicted probabilities according to the multinomial logistic regression model with disease incidence as the response variable and soil biotic and abiotic parameters as explanatory variables. Left panels: (A to C) represent the predicted probability distribution curves for each single predictor (EC, Mg and *Fusarium* population) effect fitted separately. Right panels: (D to F) represent the predicted probabilities for each of the three parameters when the other two are fixed.

To determine the relationship between the disease incidence groups of the four years of evaluation and soil abiotic and biotic parameters, a multinomial logistic regression analysis was performed. Logistic regression models fitted for each variable individually exhibited significant differences between the disease incidence classes in all abiotic and biotic parameters (pH, P, Ca, Mg, H+Al, SB, CEC, V, EC, bacteria and *Fusarium* populations), except OM and K. EC, Mg and the *Fusarium* population that were the best explanatory parameters and had the highest correct classification rates, i.e., 81.94, 83.33 and 66.67% of the cases, respectively, correctly classified (Table 1 and Fig 5A–5C).

**Table 1:** Results of multinomial logistic regression models fitted for each variable separately and multivariate multinomial logistic regression model fitted with a stepwise method.

	LRT test			
Predictor	Chi-square	P>Chi-square	Max-rescaled R^2^	Correct classification (%)
EC	157.0254	< 0.0001	0.8842	81.94
Mg	198.0991	< 0.0001	0.5837	69.44
*Fusarium*	187.3121	< 0.0001	0.4735	66.67
EC, Mg, *Fusarium*	206.8369	< 0.0001	0.9845	83.33

EC, electrical conductivity

A multiple logistic model using a stepwise procedure was fitted, selecting a model that included the three explanatory parameters (EC, Mg and the *Fusarium* population). The model explained 98.45% of the total variability and correctly classified 86.81% of the cases (Table 1 and Fig 5D–5F). The predicted probability distribution curves corresponding to the disease incidence classes from low to severely affected were distinct for all the selected parameters in the model (Fig 5D–5F). In the selected model, the probability of higher disease incidence increased with an increase in the EC, Mg and *Fusarium* population. The predicted probability distribution curves for each parameter included in the model while holding the remaining two parameters constant are shown in Fig 5D–5F. The predicted probability distribution curves corresponding to the disease incidence classes from low to severely affected were distinct for all the parameters (Fig 5). In years 3 and 4, the logistic regression models fitted for EC and N-NH_4_^+^ (data not shown) exhibited significant differences between the disease classes.

A dominant effect of the soil abiotic factors on disease incidence was observed, particularly EC. Among the biotic factors, the *Fusarium* population was the main variable responsible for the separation of disease incidence into classes. When these two main variables were compared, EC presented a dominant effect.

## Discussion

There is strong evidence throughout the literature that the incorporation of organic matter from crop waste or debris into the soil improves the soil physical properties and increases microbial activity and nutrient contents and therefore stimulates plant growth and increases soil suppressiveness to soil borne pathogens [3, 29, 47, 48]. The increase in soil suppressiveness is generally correlated with increased microbial activity resulting from the incorporation of organic matter [1]. Under the same experimental conditions as those used in the current study, Fernandes et al. [6] showed that basal respiration, microbial biomass, the metabolic quotient and enzymatic activity (urease and amylase) in the soil were positively correlated with sewage sludge dosages. However, in our study, we observed that Fusarium stalk rot increased with increasing dosages of sewage sludge applied to the soil even when the stimulation of microbial activity was considered (Figs 1–4). Thus, the annual application of higher than recommended dosages induced soil conduciveness to Fusarium stalk rot. This fact may indicate that in addition to the microbial activity, other soil attributes, which were modified by the incorporation of the sewage sludge, could also play a role in the induction of disease suppressiveness. In the present study, according to the multinomial logistic regression, the parameters with the highest explanatory power were EC, Mg and the *Fusarium* population (Fig 5, Table 1).

The EC in the soil was positively correlated with the sewage sludge concentration and the diseased plant incidence (Fig 5). In contrast, Cotxarrera et al. [16] observed that high EC was one of the factors involved in the suppression of Fusarium wilt of tomato when a sewage sludge compost was used. An opposite effect was observed by Santos and Bettiol [5], who found that increasing EC, due to the incorporation of sewage sludge, was directly correlated with an increase in soil suppressiveness to *Sclerotium rolfsii*. However, Chitarra et al. [49] concluded that an increase in the EC of the nutrient solution did not affect tomato Fusarium wilt.

A deficiency or excess of Mg can influence disease incidence because it can affect a wide range of plant physiologic functions [50]. Thus, Mg nutrition may reduce some diseases and increase others, such as Fusarium corn stalk rot in this study. The ratio between Mg and other nutrients is also of great importance for the occurrence of diseases. The synergy or antagonism among various nutrients may affect disease incidence, as verified in this study (Figs 1–3), for example, high levels of Mg could have inhibited the uptake of K, Mn, and Ca [50]. Sewage sludge has a low K content; therefore, this nutrient was supplemented in the experimental plots [24]. An insufficient supplementation could lead to an increase in the incidence of Fusarium stalk rot because low levels of K can increase disease incidence.

The association between N and plant diseases depends on the rate and timing of the application, the form of N, soil conditions, and interactions with other elements [51]. A general concept is that nitrogen frequently tends to increase disease incidence. The soil N-NH_4_^+^ and N-NO_3_^-^ contents increased during years 3 and 4 (data not shown). In addition, Fernandes et al. [52], in the same experiment, measured an increase of 59% and 66% of total N relative to the control at the highest sludge rate in the 0- to 10-cm and 10- to 20-cm layers, respectively. An extensive list of plant diseases is influenced by N-NH_4_^+^ and N-NO_3_^-^ [53], including the occurrence of diseases caused by *Fusarium* spp. in several crops. This fact is of relevance in this study because the total N content (organic, N-NH_4_^+^ and N-NO_3_^-^) available in the soil increased in successive years with repeated sludge applications.

The observed increase in disease incidence in this study can be explained in part by the nutrient imbalance caused by the sewage sludge application because this waste is not nutritionally balanced. Thus, nutritional disequilibrium caused by annual applications of high dosages of sewage sludge to the soil could be one of the factors leading to an increase in corn stalk rot incidence. High dosages of sewage sludge were applied in the present study to simulate the cumulative effect of a long-term incorporation of repeated soil application of this waste. However, higher dosages were used in others studies [11, 12, 15, 54].

The increase in soil conduciveness to Fusarium stalk rot with serial applications and high dosages of sewage sludge is in agreement with the results obtained by McIlveen and Cole Jr [18] and Millner et al. [19]. However, in general, our results differed from the compilation published by Bonanomi et al. [4] who found that in most studies there was an increase in soil suppressiveness to plant pathogens with the application of organic matter. Further, Bonanomi et al. [8] indicated that organic matter was consistently suppressive to different pathogens although few studies and a limited number of pathogens were tested. The authors added that in the majority of the studies, a material suppressive to a pathogen was ineffective or even conducive to other pathogens, suggesting that organic matter suppressiveness is often pathogen-specific and decomposition in many studies is a crucial process affecting suppressiveness. During decomposition, disease suppression might increase, decrease, remain unchanged or show more complex responses.

In this study, the bacterial populations were somewhat related to disease incidence (Figs 1 and 4). These results may be associated with the microbial pool that was introduced [55], stimulated or inhibited by the sludge. Torsvik et al. [56] showed that sludge-amended soil, without or with either low or high heavy metal contents, significantly reduced the number of bacterial species. Bacterial functional diversity is depressed in soils that are amended with high dosages of sewage sludge [57]. Soil suppressiveness, however, may be a result of a set of numerous species. More than 30,000 bacterial and archaeal species associated with *R*. *solani* suppression were detected by Mendes et al. [58]. The change in the bacterial community in the soil caused by sewage sludge might have contributed to the increased incidence of the disease in the current study.

The occurrence of diseases caused by *Fusarium* spp. is generally associated with high soil acidity. In this study, it was necessary to correct the pH of the plots treated with sewage sludge because of the high acidity of the soil. This might have masked the effect of soil pH on disease incidence. However, it is necessary to consider that the soil samples were collected 90 days after the incorporation of the sewage sludge. It is possible that shortly after the application of the sludge, acids may have been released and microsites may have become available where the roots could have been infected at the beginning of the cycle, whereas the symptoms were only manifested at the end of the crop cycle.

In general, the increase in residue dosages, including sewage sludge applied to the soil, is directly related to an increase in crop yield [59]. Thus, there is a tendency to recommend, or farmers actually use, doses that exceed the recommended values (based on the N content). However, this work clearly indicates that the increase in the incidence of Fusarium stalk rot is directly related to the increase in the sewage sludge dosage, whereas corn productivity does not respond in the same way, indicating that the disease and nutritional disequilibrium affect productivity (Figs 1–3).

The type of sewage sludge and the applied dosage may negatively or positively influence the occurrence of plant diseases. Pascual et al. [54] observed that the effect of sewage sludge on *Verticillium*-induced wilt in pepper plants was dependent on the type of sludge treatment and the dose applied. Composted sludge caused a decrease in Verticillium wilt when applied at lower doses, but the highest dose enhanced the disease, probably due to the high content of soluble salts in the compost. We recommend that the amount of sludge be calculated based on the N crop needs, application should not exceed soil capacity and the balance of other nutrients must be considered in soil fertilization in agricultural production. Agricultural use is one the best and most ecologically friendly disposal methods for sewage sludge, but all impacts should be evaluated simultaneously to prevent economic losses.

## Supporting Information

S1 Fig Dendrogram showing results of cluster analyses and heat map representation of disease, yield, and soil abiotic and biotic parameters amended with two types of sewage sludge for each of the years (1, 2, 3 and 4) individually. Dendrogram showing the results of the cluster analyses and a heat map representation of disease incidence and yield and their relationship with soil abiotic (pH, OM, P, K, Ca, Mg, H+Al, SB, CEC, V, EC, N-NH_4_^+^, N-NO_3_^-^) and biotic parameters (bacterial, fungal and *Fusarium* populations) of soil treated with sewage sludge from Franca (F) and Barueri (B) at different dosages [0N, 1N, 2N, 4N and 8N, based on the N concentration that provided the same amount of N as the mineral fertilizer (NPK) recommended for corn] for each of the years (1, 2, 3 and 4) individually. The blue intensity represents the mean value of the variable (higher values are represented by darker blue shades).
(PPTX)

S2 Fig Boxplots for soil abiotic and biotic parameters according to the three distinct groups of disease incidence obtained from the cluster analyses, from low disease incidence (group 1) to high incidence (group 3). (DOCX)

S1 Table Analysis of variance for the effects of sewage sludge from Franca and Barueri at different dosages on corn stalk rot incidence, corn yield, organic matter content, *Fusarium* and bacterial populations in soil for four years of experiments. (DOCX)

S2 Table Analysis of variance for the effects of sewage sludge from Franca and Barueri at different dosages on phosphorus, potassium, calcium, and magnesium contents, and cation exchange capacity (CEC) in soil for four years of experiments. (DOCX)

S3 Table Analysis of variance for the effects of sewage sludge from Franca and Barueri at different dosages on pH, electrical conductivity (EC), hydrogen and aluminum content, sum of bases and base saturation in soil for four years of experiments. (DOCX)
