# Supplementary material for: Combined Effects of Soil Biotic and Abiotic Factors, Influenced by Sewage Sludge Incorporation, on the Incidence of Corn Stalk Rot
Source: PLoS One. 2016 May 13;11(5):e0155536. doi: 10.1371/journal.pone.0155536 (PMC4866708; doi:10.1371/journal.pone.0155536)
Supplement: S1 Fig — Dendrogram showing the results of the cluster analyses and a heat map representation of disease incidence and yield and their relationship with soil abiotic (pH, OM, P, K, Ca, Mg, H+Al, SB, CEC, V, EC, N-NH4+, N-NO3-) and biotic parameters (bacterial, fungal and Fusarium populations) of soil treated with sewage sludge from Franca (F) and Barueri (B) at different dosages [0N, 1N, 2N, 4N and 8N, based on the N concentration that provided the same amount of N as the mineral fertilizer (NPK) recommended for corn] for each of the years (1, 2, 3 and 4) individually. The blue intensity represents the mean value of the variable (higher values are represented by darker blue shades). (PPTX) [file pone.0155536.s001.pptx]

## Slide 1
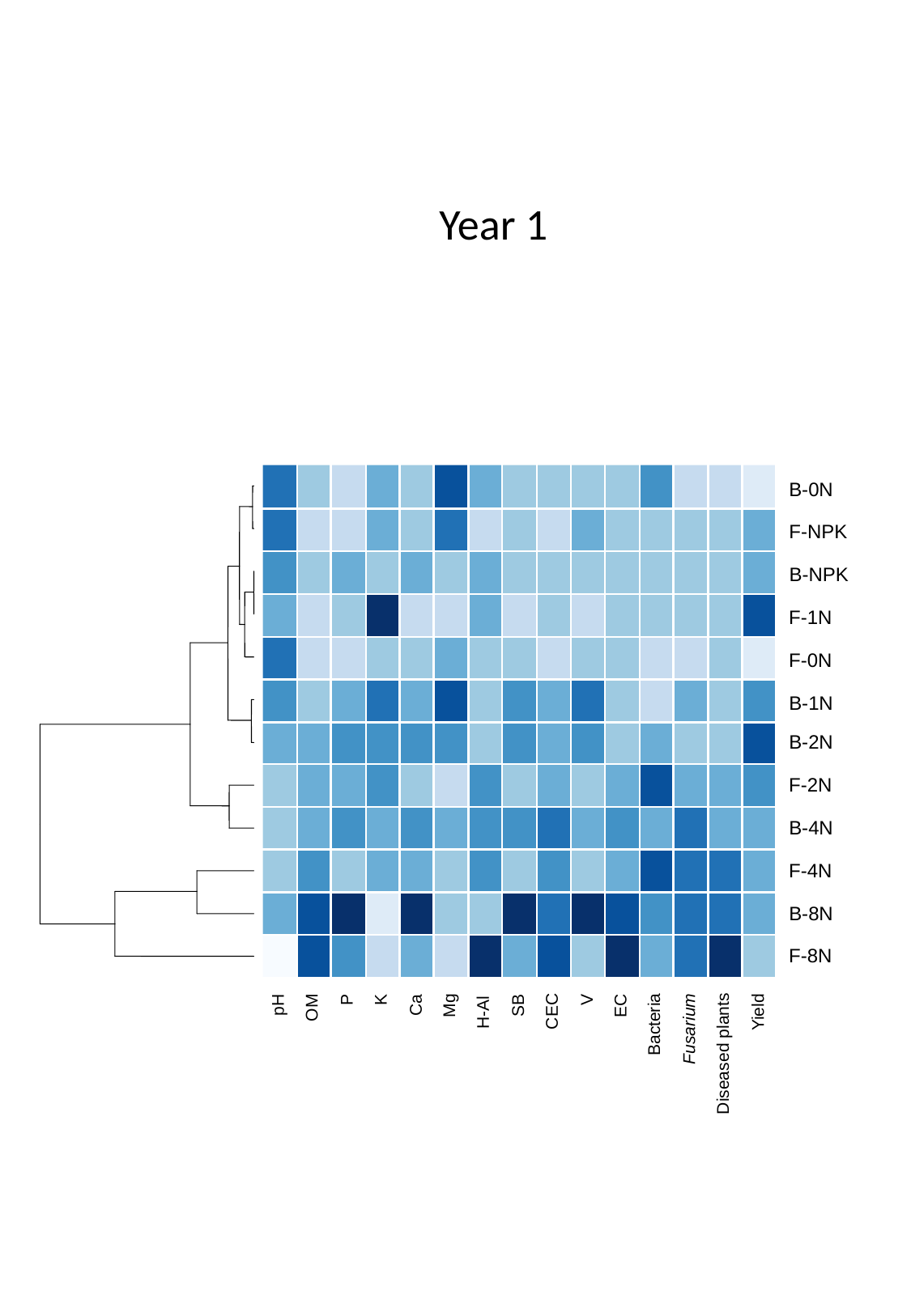

Year 1
B-0N
F-NPK
B-NPK
F-1N
F-0N
B-1N
B-2N
F-2N
B-4N
F-4N
B-8N
F-8N
P
K
V
pH
Ca
SB
Mg
EC
OM
CEC
Yield
H-Al
Bacteria
Fusarium
Diseased plants

## Slide 2
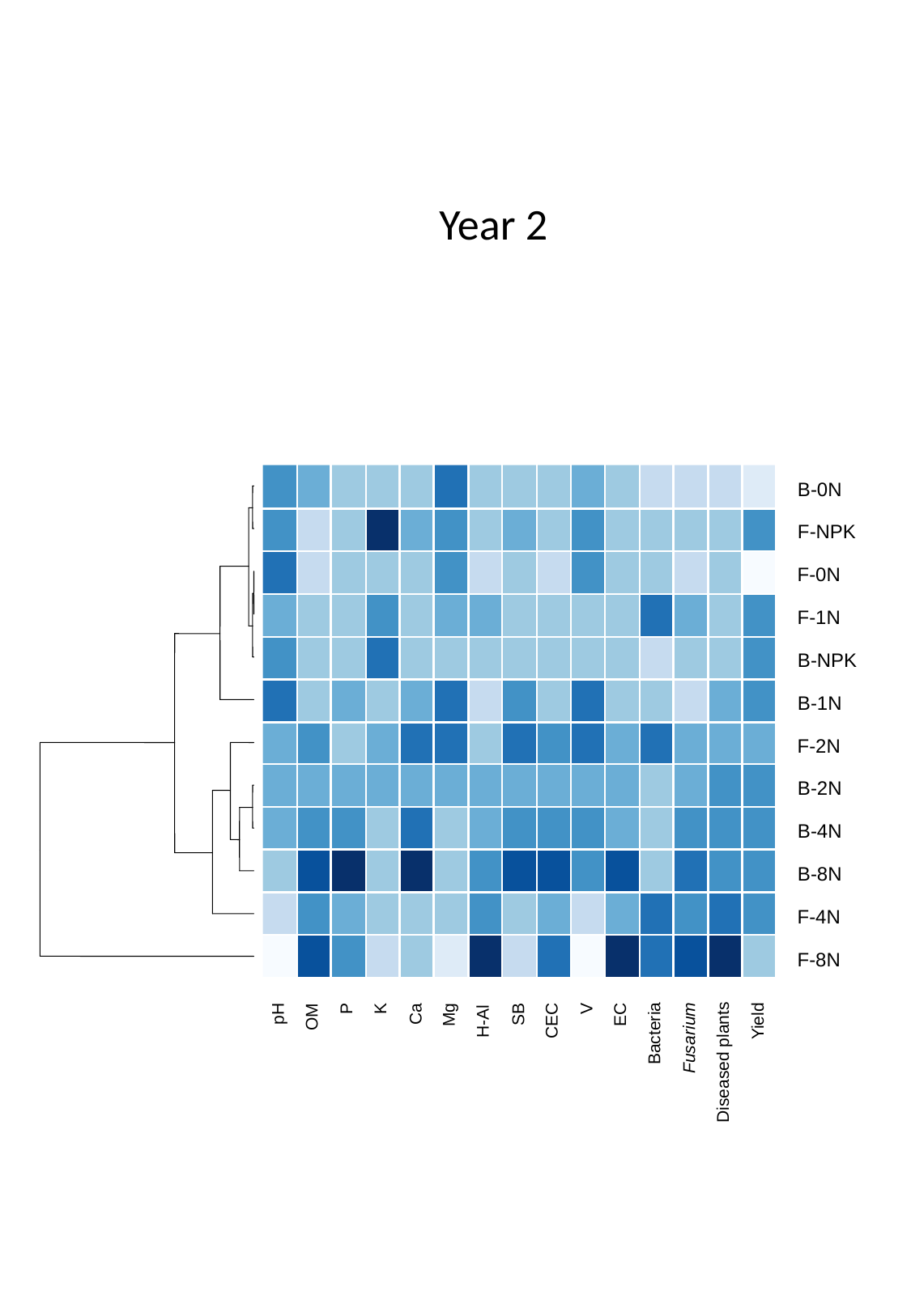

Year 2
B-2N
B-4N
B-8N
F-4N
F-8N
P
K
V
pH
Ca
SB
Mg
EC
OM
CEC
H-Al
Yield
Bacteria
Fusarium
Diseased plants
B-0N
F-NPK
F-0N
F-1N
B-NPK
B-1N
F-2N

## Slide 3
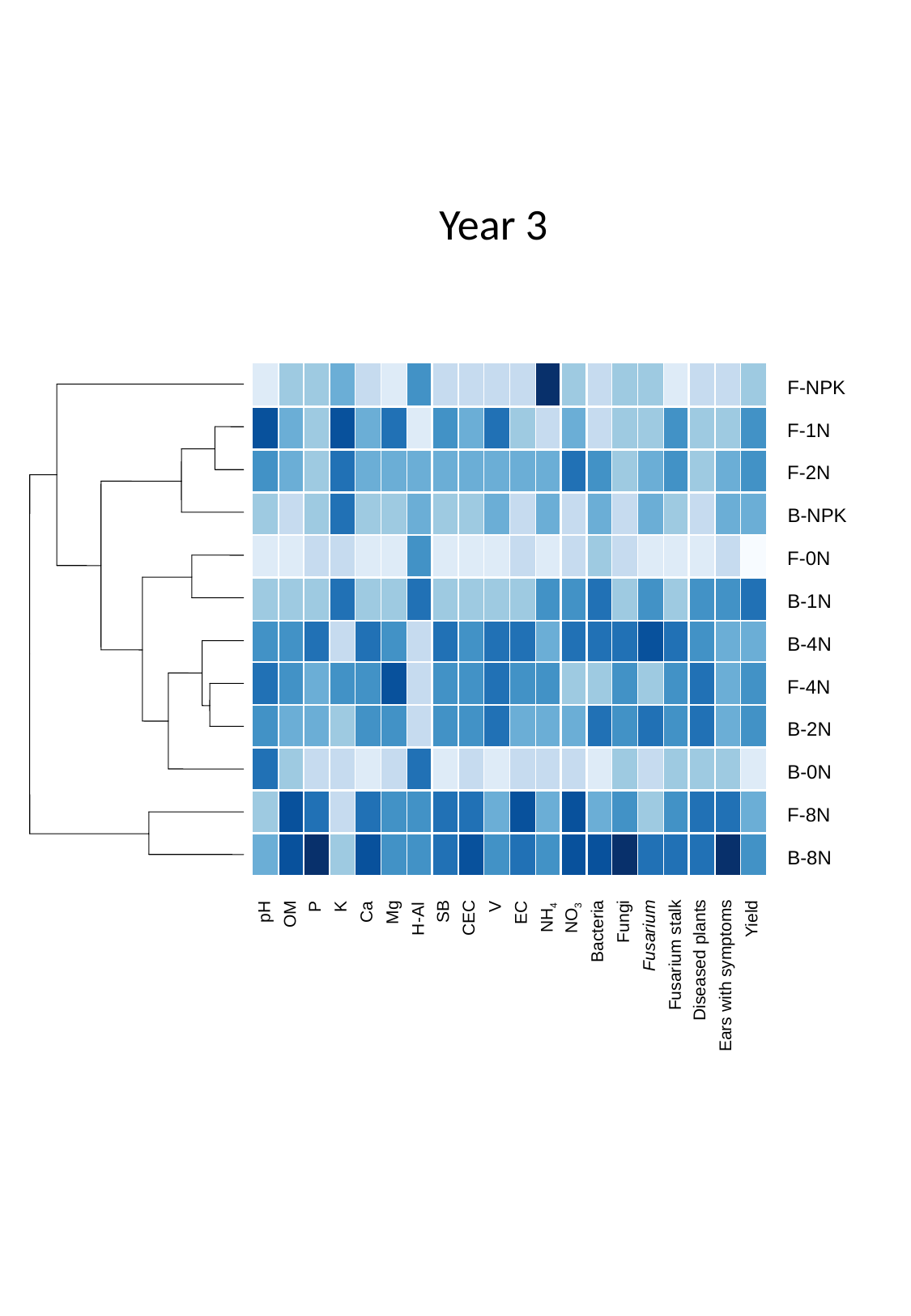

Year 3
F-NPK
F-1N
F-2N
B-NPK
F-0N
B-1N
B-4N
F-4N
B-2N
B-0N
F-8N
B-8N
P
K
V
pH
Ca
SB
Mg
EC
OM
NH4
CEC
NO3
Yield
H-Al
Fungi
Bacteria
Fusarium
Fusarium stalk
Diseased plants
Ears with symptoms

## Slide 4
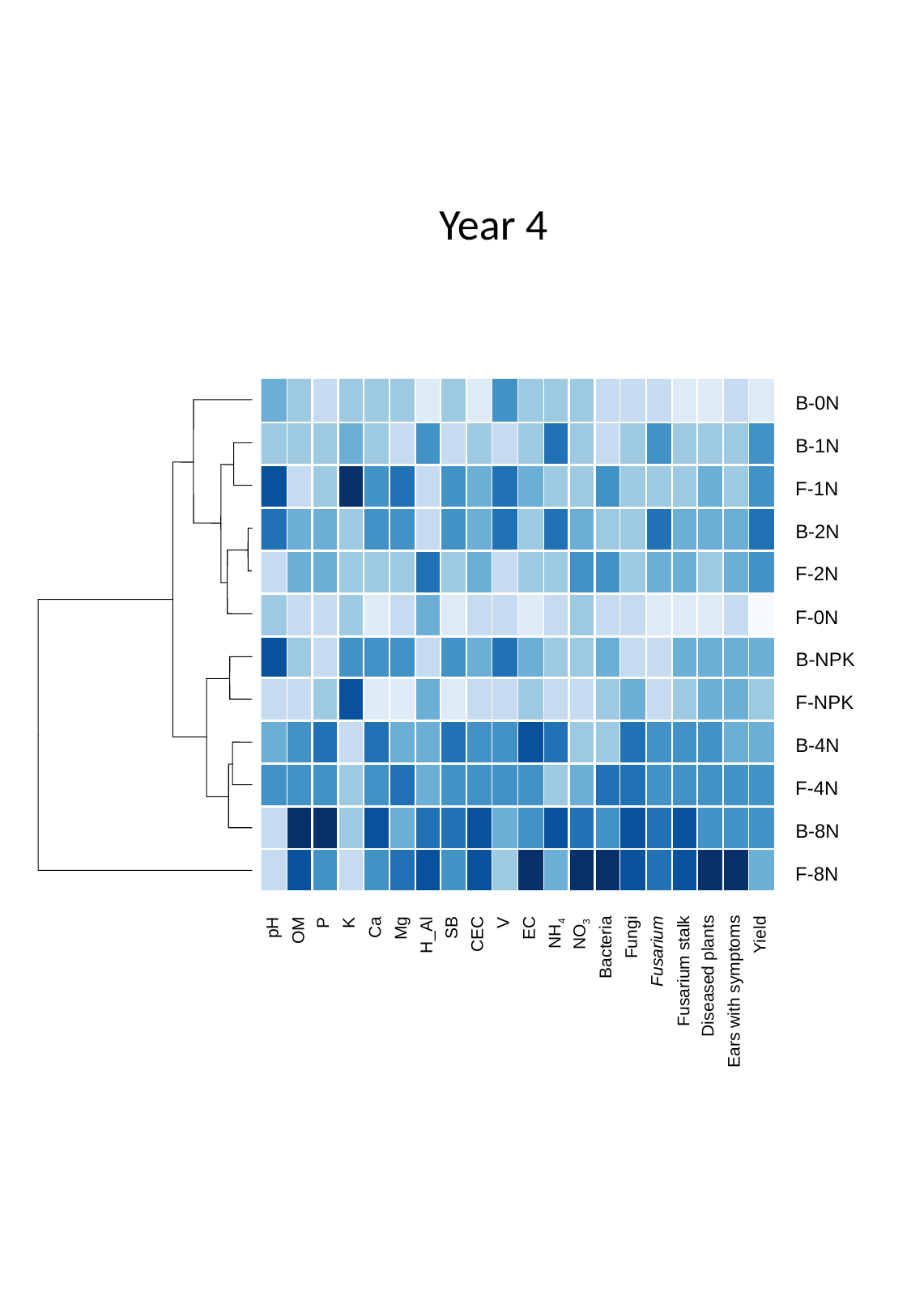

Year 4
B-0N
B-1N
F-1N
B-2N
F-2N
F-0N
B-NPK
F-NPK
B-4N
F-4N
B-8N
F-8N
P
K
V
pH
Ca
SB
Mg
EC
OM
NH4
CEC
NO3
H_Al
Yield
Fungi
Bacteria
Fusarium
Fusarium stalk
Diseased plants
Ears with symptoms
