# Supplementary material for: Combined Effects of Soil Biotic and Abiotic Factors, Influenced by Sewage Sludge Incorporation, on the Incidence of Corn Stalk Rot
Source: PLoS One. 2016 May 13;11(5):e0155536. doi: 10.1371/journal.pone.0155536 (PMC4866708; doi:10.1371/journal.pone.0155536)
Supplement: S1 Table — (DOCX) [file pone.0155536.s003.docx]

**S1 Table.** Analysis of variance for the effects of sewage sludge from Franca and Barueri at different dosages on corn stalk rot incidence, corn yield, organic matter content, *Fusarium* and bacterial populations in soil for four years of experiments.

| **Between Years effects** | | | | | | | |  |  | | |  |  | | |  |  | | |
| --- | --- | --- | --- | --- | --- | --- | --- | --- | --- | --- | --- | --- | --- | --- | --- | --- | --- | --- | --- |
|  | Stalk rot incidence | | |  | Corn yield | | |  | Organic matter content | | |  | *Fusarium* population | | |  | Bacterial population | | |
| Source | df | *F* | *P* |  | df | *F* | *P* |  | df | *F* | *P* |  | df | *F* | *P* |  | df | *F* | *P* |
| Sewage (S) | 1 | 0.92 | 0.3476 |  | 1 | 4.95 | 0.0358 |  | 1 | 1.77 | 0.1959 |  | 1 | 0.42 | 0.5209 |  | 1 | 9.04 | 0.0061 |
| Dose (D) | 5 | 55.94 | <0.0001 |  | 5 | 29.65 | <0.0001 |  | 5 | 28.78 | <0.0001 |  | 5 | 45.04 | <0.0001 |  | 5 | 3.92 | 0.0097 |
| S x D | 5 | 4.59 | 0.0044 |  | 5 | 1.13 | 0.3694 |  | 5 | 0.99 | 0.4471 |  | 5 | 0.22 | 0.9509 |  | 5 | 1.41 | 0.2548 |
| **Within Years effects** | | | | | | |  |  |  |  |  |  |  |  |  |  |  |  |  |
|  | Stalk rot incidence | | |  | Corn yield | | |  | Organic matter content | | |  | *Fusarium* population | | |  | Bacterial population | | |
| Source | df | *F* | *P* |  | df | *F* | *P* |  | df | *F* | *P* |  | df | *F* | *P* |  | df | *F* | *P* |
| Year (Y) | 3 | 356.18 | <0.0001 |  | 3 | 11.42 | <0.0001 |  | 3 | 137.96 | <0.0001 |  | 3 | 177.30 | <0.0001 |  | 3 | 56.04 | <0.0001 |
| Y x S | 3 | 8.71 | 0.0012 |  | 3 | 0.76 | 0.5216 |  | 3 | 1.18 | 0.3229 |  | 3 | 5.26 | 0.0288 |  | 3 | 3.15 | 0.0593 |
| Y x D | 15 | 4.37 | 0.0006 |  | 15 | 2.49 | 0.0053 |  | 15 | 0.65 | 0.8202 |  | 15 | 16.61 | <0.0001 |  | 15 | 0.97 | 0.4742 |
| Y x S x D | 15 | 1.23 | 0.3050 |  | 15 | 0.85 | 0.6155 |  | 15 | 0.48 | 0.9425 |  | 15 | 0.55 | 0.7483 |  | 15 | 0.33 | 0.9569 |
