# Supplementary material for: Combined Effects of Soil Biotic and Abiotic Factors, Influenced by Sewage Sludge Incorporation, on the Incidence of Corn Stalk Rot
Source: PLoS One. 2016 May 13;11(5):e0155536. doi: 10.1371/journal.pone.0155536 (PMC4866708; doi:10.1371/journal.pone.0155536)
Supplement: S3 Table — (DOCX) [file pone.0155536.s005.docx]

**S3 Table.** Analysis of variance for the effects of sewage sludge from Franca and Barueri at different dosages on pH, electrical conductivity (EC), hydrogen and aluminum content, sum of bases and base saturation in soil for four years of experiments.

| **Between Years effects** | | | | | | | |  |  | | |  |  | | |  |  | | |
| --- | --- | --- | --- | --- | --- | --- | --- | --- | --- | --- | --- | --- | --- | --- | --- | --- | --- | --- | --- |
|  | pH | | |  | EC | | |  | Hydrogen and aluminum | | |  | Sum of bases | | |  | Base saturation | | |
| Source | df | *F* | *P* |  | df | *F* | *P* |  | df | *F* | *P* |  | df | *F* | *P* |  | df | *F* | *P* |
| Sewage (S) | 1 | 6.75 | 0.0158 |  | 1 | 1.76 | 0.1958 |  | 1 | 4.44 | 0.0457 |  | 1 | 15.60 | 0.0006 |  | 1 | 14.28 | 0.0009 |
| Dose (D) | 5 | 5.58 | 0.0015 |  | 5 | 216.59 | <0.0001 |  | 5 | 9.97 | <0.0001 |  | 5 | 18.95 | <0.0001 |  | 5 | 2.78 | 0.0404 |
| S x D | 5 | 1.33 | 0.2837 |  | 5 | 10.32 | <0.0001 |  | 5 | 3.18 | 0.0241 |  | 5 | 2.21 | 0.0860 |  | 5 | 2.42 | 0.0649 |
| **Within Years effects** | | | | | | |  |  |  |  |  |  |  |  |  |  |  |  |  |
|  | pH | | |  | EC | | |  | Hydrogen and aluminum | | |  | Sum of bases | | |  | Base saturation | | |
| Source | df | *F* | *P* |  | df | *F* | *P* |  | df | *F* | *P* |  | df | *F* | *P* |  | df | *F* | *P* |
| Year (Y) | 3 | 16.44 | <0.0001 |  | 3 | 734.87 | <0.0001 |  | 3 | 138.73 | <0.0001 |  | 3 | 112.88 | <0.0001 |  | 3 | 129.70 | <0.0001 |
| Y x S | 3 | 0.37 | 0.7767 |  | 3 | 2.95 | 0.0738 |  | 3 | 1.48 | 0.2392 |  | 3 | 0.59 | 0.6213 |  | 3 | 1.44 | 0.2488 |
| Y x D | 15 | 3.14 | 0.0006 |  | 15 | 50.48 | <0.0001 |  | 15 | 2.03 | 0.0670 |  | 15 | 2.79 | 0.0019 |  | 15 | 1.85 | 0.1006 |
| Y x S x D | 15 | 2.95 | 0.0011 |  | 15 | 2.67 | 0.0186 |  | 15 | 2.63 | 0.0203 |  | 15 | 2.16 | 0.0160 |  | 15 | 2.97 | 0.0123 |
