# Supplementary material for: Combined Effects of Soil Biotic and Abiotic Factors, Influenced by Sewage Sludge Incorporation, on the Incidence of Corn Stalk Rot
Source: PLoS One. 2016 May 13;11(5):e0155536. doi: 10.1371/journal.pone.0155536 (PMC4866708; doi:10.1371/journal.pone.0155536)
Supplement: Supplementary file 5 [file pone.0155536.s002.docx]

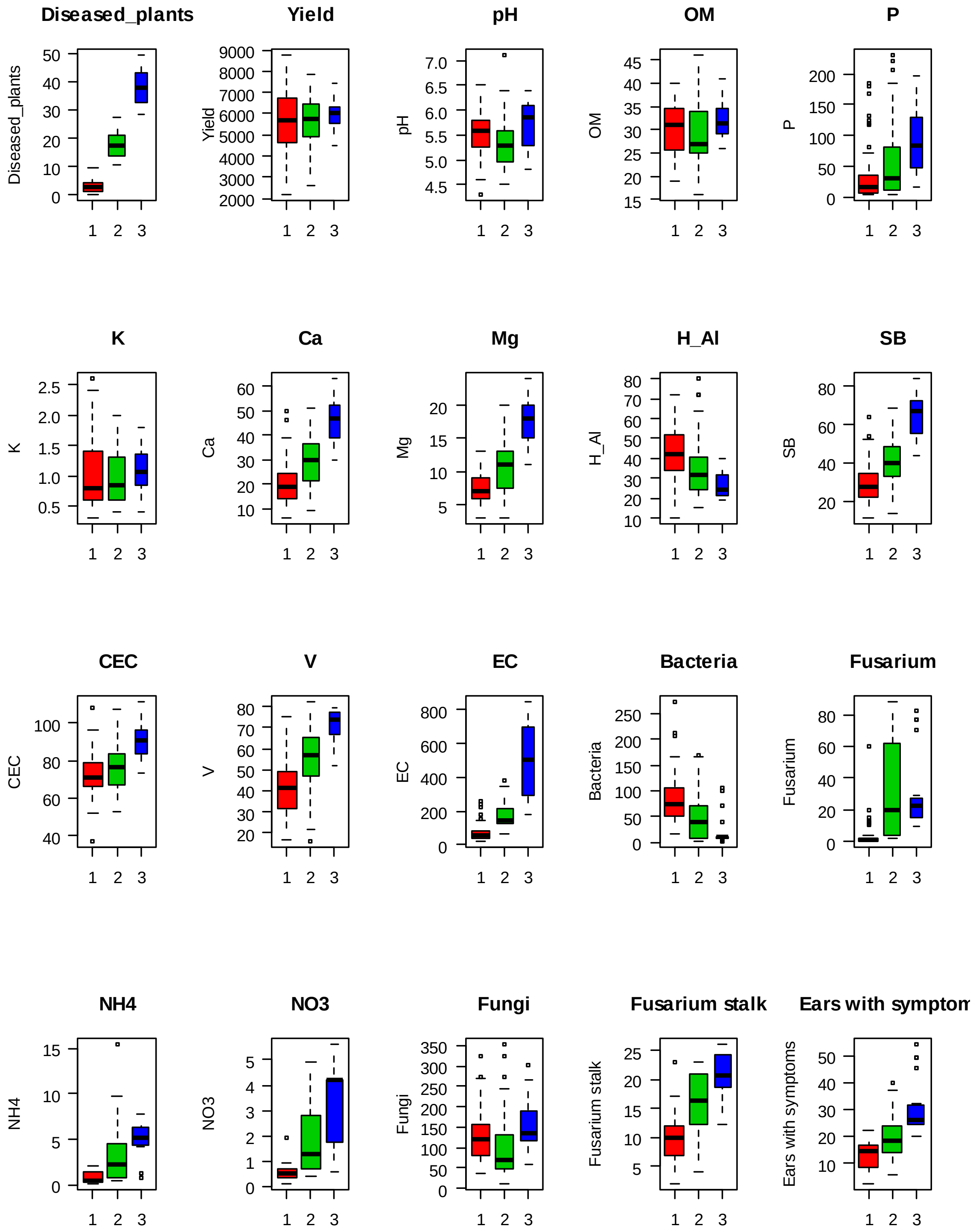
Diseased_plants
Yield
pH
OM
P
K
Ca
Mg
H_Al
SB
CEC
V
EC
Bacteria
Fusarium
NH4
NO3
Fungi
Fusarium stalk
Ears with sympto
Ears with symptoms
1 2 3
